# Effects of exercise intervention on tobacco dependence: a meta-analysis

**DOI:** 10.3389/fpubh.2025.1538833

**Published:** 2025-02-18

**Authors:** Jinzhi Xu, Shiyue Zhang, Zichao Chen, Zhusheng Wu

**Affiliations:** ^1^Department of Physical Education, Qufu Normal University, Qufu, China; ^2^School of Physical Education, Sichuan University, Chengdu, China; ^3^School of Tourism, Sichuan University, Chengdu, China

**Keywords:** tobacco dependence, physical exercise, acute aerobic exercise, smoking cessation, meta-analysis

## Abstract

**Introduction:**

Smoking poses a significant threat to global human health, making smoking cessation a controllable means of preventing mortality. Exercise, as a means of promoting a healthy lifestyle, offers substantial benefits to individuals attempting to quit smoking. However, due to variations in experimental populations and conditions, the specific effects and benefits of exercise on smoking cessation remain unclear. In this meta-analysis, we comprehensively evaluated the withdrawal effects of different intensities of exercise on tobacco-dependent individuals.

**Methods:**

Statistical analysis and graphing were performed using Stata 14 and Review Manager 5.4 software. A total of 47 literatures, encompassing 57 randomized controlled trials and involving 4,267 tobacco-dependent individuals, were included.

**Results:**

The meta-analysis results showed that long-term exercise had no significant difference or impact on the degree of tobacco dependence between the exercise and control groups. However, acute exercise was associated with increased tobacco craving (desire and intensity) and more pronounced withdrawal symptoms.

**Discussion:**

Acute aerobic exercise can significantly reduce craving and withdrawal symptoms among individuals attempting to quit smoking, demonstrating a certain role in smoking cessation. Acute aerobic exercise emerges as the most effective form of physical exercise for intervening in tobacco dependence.

**Systematic review registration:**

https://www.crd.york.ac.uk/PROSPERO/, CRD42024550014.

## 1 Introduction

According to the World Health Organization (WHO) report, approximately 8 million people globally die from tobacco-related causes annually, with 7 million deaths attributed directly to smoking and around 1.2 million to exposure to secondhand smoke (1). Smoking, as a significant factor contributing to the high incidence of malignancies, cardiovascular and cerebrovascular diseases, respiratory diseases, diabetes, and other illnesses, poses a severe threat to human health (2). Furthermore, studies have shown that during the COVID-19 pandemic, smokers had a higher mortality rate due to viral pneumonia compared to non-smokers (3).

Therefore, addressing the difficulty of smoking cessation among smokers has become an issue worthy of in-depth exploration. There are various ways to assist in smoking cessation, such as psychological intervention, self-regulation, pharmacotherapy, and food substitution (4). Although these methods have certain effects on smoking cessation, they cannot meet the goal of long-term abstinence, and the results are not significant (5). For example, psychological intervention and pharmacotherapy may have certain dependency and side effects, making it difficult for smokers to quit smoking independently (6). Food substitution and other methods may lead to overeating and cause physical harm (7).

Through reviewing related literatures, it is known that exercise can effectively help smokers reduce smoking frequency, alleviate withdrawal symptoms, improve cardiopulmonary function, reduce anxiety about weight gain, and obtain social support and encouragement (8–10). This is because exercise releases chemicals such as dopamine by increasing neurotransmitter regulation, thereby alleviating withdrawal symptoms during the smoking cessation process (11). At the same time, exercise, as a positive alternative behavior, can replace smoking, which is a negative behavior (12). However, due to variations in exercise intensity and frequency, academic research on the effectiveness of exercise interventions for smoking cessation has yet to yield definitive conclusions.

The necessity of studying the impact of exercise interventions on tobacco dependence is further underscored by previous research, such as the reviews conducted by Zhou et al. (13), Santos et al. (14) and Klinsphone et al. (9), which examined the effect of exercise interventions on smoking cessation. However, despite these efforts, several gaps remain in our understanding of this complex relationship. First, some studies have analyzed single or scattered outcome indicators, limiting our comprehensive understanding of the effects of exercise. For instance, previous research focused on smoking cessation rates and mood, but relatively overlooked other potential indicators such as sleep quality. Second, subgroup analyses were not sufficiently detailed or comprehensive. Previous research primarily examined the effects on smoking cessation rates and smoking cravings (13), neglecting other important aspects that could further identify differential factors. For example, the differential effects of exercise on depression among smokers have not been thoroughly explored. A more nuanced approach to subgroup analysis is essential for gaining a deeper understanding of how exercise interventions can impact tobacco dependence. Furthermore, the quality of studies included in previous meta-analyses has been deemed suboptimal (9, 13), potentially affecting the credibility of the results. Improving the overall quality of research in this field is crucial, and one way to achieve this is by increasing the sample size. In light of these limitations, the present meta-analysis aims to further explore and analyze the topic of exercise interventions on tobacco dependence, building upon previous systematic reviews. By including more studies and focusing on a more comprehensive set of outcome indicators, this study seeks to address the gaps identified in previous research.

In summary, despite existing research on the effects of exercise interventions on tobacco dependence, there remains a need for a more comprehensive and nuanced understanding of this relationship. The present study contributes to this understanding by conducting a meta-analysis that includes a larger number of studies, employs stricter screening and inclusion criteria, selects more comprehensive outcome indicators, and performs detailed subgroup analyses according to various indicators.

## 2 Methods and materials

This study was conducted according to the latest PRISMA (Preferred Reporting Items for Systematic Reviews and Meta-Analyses) guidelines for reporting systematic reviews (15). The study has been registered on the Prospero website (Registration Number: CRD42024550014).

### 2.1 Inclusion and exclusion criteria

#### 2.1.1 Inclusion criteria

(1) Study Type: randomized controlled trials (RCTs), regardless of whether blinding was used. The language was limited to English.
(2) Population: Adult patients with a history of smoking (excluding pregnant women) who met the diagnostic criteria for tobacco dependence according to the “Chinese Clinical Guidelines for Smoking Cessation 2015.”
(3) Interventions: The experimental group received interventions with varying intensities of exercise for smoking cessation; the control group received adjuvant therapies such as health education lectures, reading, or sitting quietly. Other adjuvant means related to smoking cessation were consistent between the two groups.
(4) Outcome Indicators: Based on the tobacco withdrawal symptoms outlined in the “Chinese Clinical Guidelines for Smoking Cessation 2015,” the outcome indicators were divided into nine categories. Smoking cessation rates: 7-day smoking cessation rate and continuous smoking cessation rate; smoking craving: Desire to Smoke (Dts) and Strength of Desire to Smoke (SoD); withdrawal symptoms: sleep quality, depression, irritability; emotions: negative emotions and positive emotions.

#### 2.1.2 Exclusion criteria

(1) Studies involving pregnant women, lactating women, or patients with other conditions that could affect smoking cessation.
(2) Studies where data could not be accurately extracted or where data were missing.
(3) Studies with inconsistent adoption of data indicators.
(4) Duplicate publications.
(5) Studies that were not focused on smoking dependence.
(6) Studies with non-randomized or semi-randomized control designs.
(7) Studies where the experimental group did not involve exercise interventions.

### 2.2 Literature search

**Figure 1:**
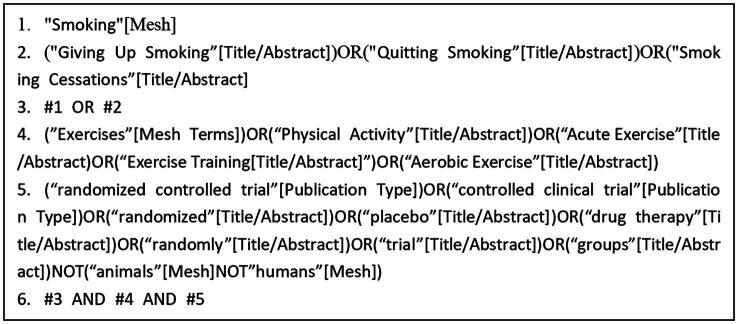
PubMed search strategy.

A computer-based search was conducted across the databases Web of Science, PubMed, The Cochrane Library, Embase, and Scopus to collect RCTs on exercise interventions for tobacco dependence. The search period covered from the inception of each database to May 2024. The search strategy combined the use of subject headings and free-text terms. Additionally, the references of the included studies were traced to supplement the acquisition of relevant literature. The search terms included: “Smoking,” “Exercises,” “Cessation,” “Acute Exercise,” and other relevant keywords (Figure 1).

### 2.3 Literature screening and data extraction

The literature search records were documented using EndNotes software. The literature retrieved from the databases was imported into EndNotes for further merging and screening to identify duplicate articles. Two researchers independently conducted the article screening process by initially examining the titles, abstracts, and keywords. In cases of disagreement, the researchers resolved the issues through consultation or discussion with a third party. The extracted information primarily included the publication year, authors, number of subjects, and basic information of the control group (age, gender, sample size, tobacco dependence level). Additionally, the exercise plans during the intervention process for both the experimental and control groups were extracted (type of exercise, duration of exercise, frequency of exercise, exercise duration, and exercise intensity). The outcome indicators for both groups before and after the intervention (mean values, standard deviations) and literature quality assessment information were also recorded.

### 2.4 Risk of bias assessment

The Review Manager software version 5.4 was used to conduct a risk of bias assessment among included studies. The evaluation items included: ① bias arising from the randomization process; ② bias due to deviations from intended interventions (effectiveness of intervention allocation); ③ bias in outcome measurement; and ④ bias in selective reporting of results. The evaluation results were categorized as “low risk,” “some concerns,” and “high risk.” Two evaluators independently conducted the methodological quality evaluation, and in cases of disagreement, consensus was reached based on a third-party opinion.

Utilizing the Cochrane “Risk of Bias” tool, the quality of the included RCTs was assessed based on seven criteria: random sequence generation, allocation concealment, blinding of participants and personnel, blinding of outcome assessment, completeness of outcome data, selective reporting of results, and other potential sources of bias. The research quality was classified into three levels: high, moderate, and low. The assessment of bias risk was independently conducted by two review authors, and in cases of disagreement, consensus was reached based on a third-party opinion.

### 2.5 Statistical analysis

The Review Manager software version 5.4 and Stata 14 were used for meta-analysis and data synthesis. The study data included dichotomous variables and continuous variables. For dichotomous variables (smoking cessation rates), the relative risk (RR) with a 95% confidence interval was summarized. For continuous variables (craving for tobacco, withdrawal symptoms, and mood), if the outcome measures and units were consistent, the weighted mean difference (WMD) was used; if different measurement methods and units were used, the standardized mean difference (SMD) was reported (16), with a 95% CI. Heterogeneity among study results was tested using *I*^2^, where *I*^2^ values of 25, 50, and 75% were considered as thresholds for low, moderate, and high heterogeneity, respectively. A fixed-effects model was selected for analysis when *I*^2^ was low (<50%); otherwise, a random-effects model was used. Sensitivity analysis was conducted by excluding trials with a risk of bias to evaluate the stability of the meta-analysis results. Additionally, subgroup analysis was employed to identify sources of heterogeneity and assess whether various factors influenced the effect size estimates. When more than 10 studies were included for an outcome measure, funnel plots and Egger’s test were used to detect publication bias.

## 3 Results

### 3.1 Results of literature screening and data extraction

**Figure 2:**
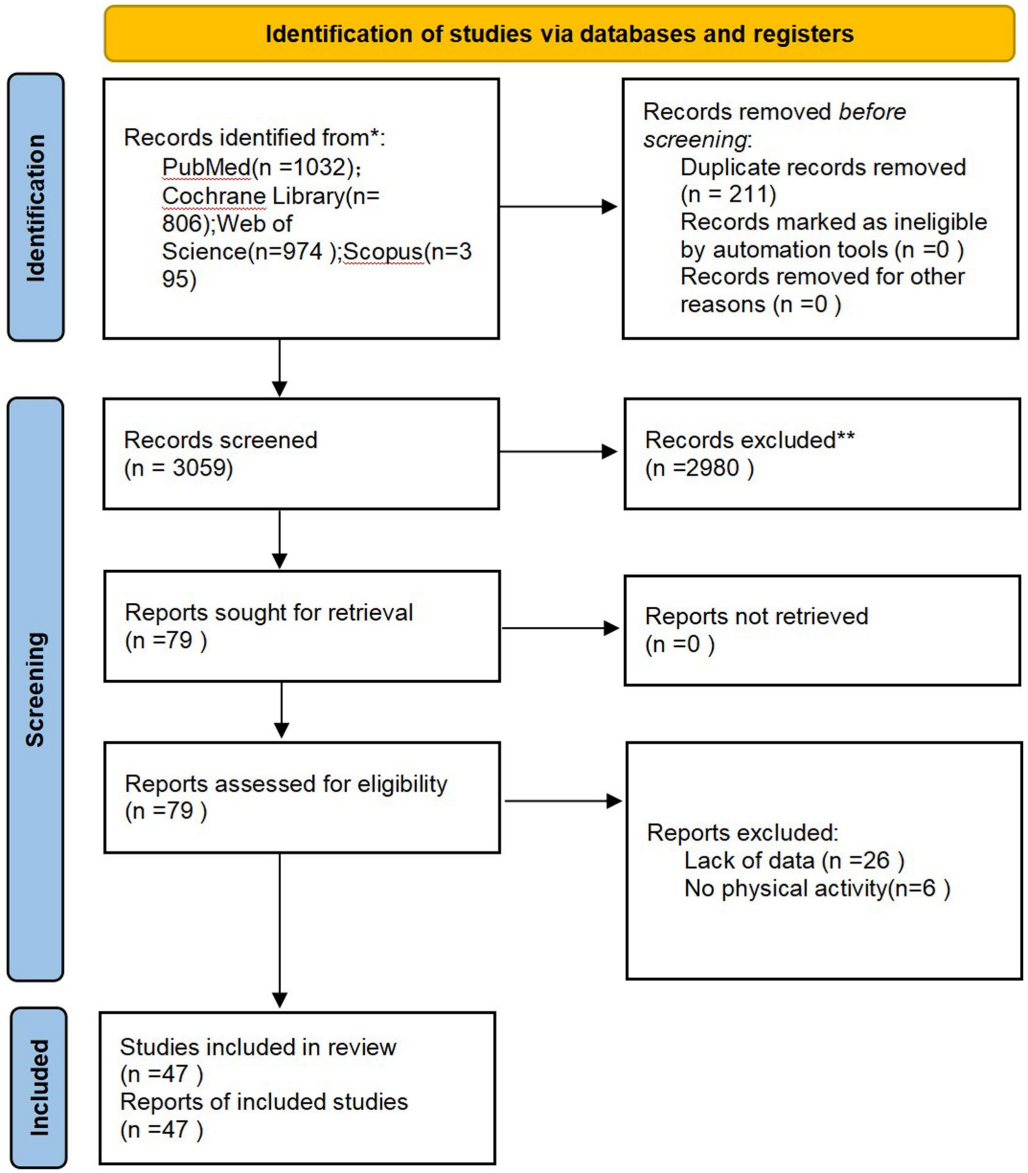
Inclusion process of literature selection.

A total of 3,207 literatures related to this topic were retrieved from various databases. After removing duplicates, screening by reading titles and abstracts, and excluding articles that were not randomized controlled trials, not related to smoking cessation, or did not involve exercise interventions, 78 articles remained. Upon reading the full texts, six articles were excluded due to intervention methods in the experimental group not involving exercise and 26 articles due to lack of available data. Finally, 47 studies were included in the study (the inclusion process is illustrated in Figure 2).

### 3.2 Characteristics of included studies

Table 1 provides an overview of the included studies. These 47 studies comprised a total of 57 RCTs, with five studies each including two RCTs. The meta-analysis included a total of 4,267 smokers. Among them, 10 studies focused solely on women, two studies did not specify the gender of participants, and 35 studies included both men and women. The age range of the participants in the experimental groups was between 18 and 65 years. The types of exercise included in these studies were aerobic exercises (such as cycling, walking, etc.), isometric exercises, yoga, walking, multi-component training, etc. Among them, 17 studies involved aerobic exercises ranging in duration from 0 to 45 min, which was the most common type of exercise.

**Table 1:** Overview of included studies.

Study	Author	Year	Sample (M/W)	Age M ± SD	Degree of tobacco dependence	Exercise schedule					Exercise			Control group intervention				Outcome indicator
						Type	Min/per session	Frequency	Duration	Intensity	N1	M	SD	N2	Type	M	SD	
1	Bock	2012	55 (0/55)	45.6 ± 8.3	5.0 ± 1.4	Yoga	45 min	2 times/wk	8 weeks	-	32	Events: 13	Total: 32	23	Watch video	Events: 3	Total: 23	A1
												−1.1	5.755867			0	7.251896	D2
2	Taylor	2007	60 (26/34)	28.3 ± 7.4	3.47 ± 2.23	Walk on treadmill	15 min	–	–	RPE 10.9 ± 1.4	31	−2.19	1.763973	29	Sitting passively	0.38	1.285574	B1
												−1.21	1.578068			0.58	1.400107	B2
												−0.35	0.72519			0.61	0.945727	C5
												−0.39	0.545802			0.3	0.987472	C4
3	Marcus	2005	217 (0/217)	42.77 ± 10.34	4.85 ± 2.32	Aerobic exercise	30–45 min	5 days/wk	8 weeks	45–59% HRR	109	Events: 22	Total: 109	108	Health education	Events: 20	Total: 108	A1
												Events: 26	Total: 134			Events: 15	Total: 147	A2
4	Al-Chalabi	2008	40 (19/21)	34.9 ± 11.7	5.2 ± 2.3	Isometric exercise		–	4 weeks	–	20	Event: 9	Total: 20	20	Sitting passively	Event: 11	Total: 20	A1
5	Abrantes	2014	61 (21/40)	47.3 ± 9.6	5.7 ± 1.9	Aerobic exercise	20–30 min	2–4 times/wk	12 weeks	55–69% HRmax	30	Events: 12	Total: 30	31	Health education	Events: 7	Total: 31	A1
												Events: 9	Total: 30			Events: 8	Total: 31	A2
												0.5	0.8544			0.2	0.8	D2
												−0.1	0.264575			−0.1	0.43589	D1
6	Ussher	2006	60 (33/27)	32.19 ± 8.94	3.9 ± 2.12	Isometric exercise	5 min	–	–	–	20	−0.95	1.906384	20	Sitting passively	−0.27	1.811325	B2
												−0.65	1.793683			−0.33	1.540876	C5
												−0.1	1.124678			−0.12	1.10218	C4
7	Abrantes	2017	118 (41/77)	-	-	aerobic exercise	20 min	3 days/week	12-weeks.	–	57	2.01	9.370011	61	Health-education	1.6	2.150279	D2
												−4.8	8.072794			1.2	7.302055	D1
8	Taylor	2005	15 (10/5)	25.6 ± 6.5	4.0 ± 3.1	Self-paced walking	15–20 min	–	–	RPE 10.8 ± 1.49	15	−4.9	1.17	15	Sitting passively	−0.7	1.33	B1
												−4.5	1.216553			0.1	1.252996	B2
9	Van Rensburg	2008	23 (15/8)	23.1 ± 4.6	3.4 ± 2.03	Walk on treadmill	15 min	–	–	RPE 10.8 ± 1.67	–	−0.8	1.31114877	–	Sitting passively	0.3	1.1031319	B1
10	De Jesus,	2018	110 (56/54)	33.41 ± 14.13	4.61 ± 1.95	Treadmill exercise	10 min	–	–	40–68% HRR	51	−1.36	1.938143	48	Sitting passively	−0.44	1.713505	B1
11	HILL	1993	82 (39/43)	50+	6.5 ± 1.6	Aerobic exercise	15–35 min	1–3 times/wk	12 weeks	60–70% HRR	38	Events: 6	Total: 18	44	Behavioral training	Events: 10	Total: 22	A1
12	Cheung	2020	208 (156/52)	40.2 ± 9.9	–	Isometric exercise	–	–	14 weeks	–	108	Events: 51	Total: 108	100	Health education	Events: 47	Total: 100	A1
13	HILL	1985	36 (10/26)	25–50	-	Aerobic exercise	30 min	2 times/wk	5 weeks	–	18	Events: 12	Total: 18	18	Group counseling	Events: 8	Total: 18	A1
14	Ussher	2001	78 (36/42)	36.6 ± 10.9	6.1 ± 2.3	Stationary cycling	10 min	–	–	40–60% HRR	42	−4.29	1.03	36	Sitting passively, watch video	0.07	0.8	B1
												−4.5	1.1			0.98	0.19	B2
15	Fong	2014	25 (11/14)	37.5 ± 14.8	3.84 ± 2.36	Treadmill exercise	15 min	–	–	45-68%HRR	12	−2.09	1.815296	13	Sitting passively	0	1.804079	B2
16	Ussher	2009	48 (31/17)	27.8 ± 8.4	5.0 ± 2.2	Isometric exercise	10 min	–	–	–	14	−1.79	1.393879	32	Read, body scanning	−1.36	1.504028	B2
												−1.72	1.477329			−0.77	1.563873	C5
17	Scerbo	2010	18 (10/8)	26.0 ± 4.2	4.4 ± 1.7	Walking/Running	15 min	–	–	(1) 45–50% HRR	36	(1)-1.9	2.223196	14	Sitting passively	−0.2	2.03973	B1
										(2) 80–85% HRR		(2)-2.2	2.20411			−0.2	2.03973	
										(1) 45–50% HRR		(1) -2	1.526204			−0.6	1.245913	B2
										(2) 80–85% HRR		(2)-2.4	1.665323			−0.6	1.756417	
18	Taylor	2006	15 (10/5)	25.6 ± 6.5	4.0 ± 3.1	Walking on treadmill	15–20 min	–	–	RPE 10.8 ± 1.49	–	−4.9	1.17	–	Sitting passively	−0.7	1.33	B1
19	Dunsiger	2021	105 (0/105)	42.5 ± 11.2		Aerobic exercise	50 min	3 times/wk	12 weeks	64–76% HRmax	58	Events: 13	Total: 52	55	View video	21	53	A1,
												Events: 7	52			9	535	A2
20	Kinnunen	2008	182 (0/182)	38.4 ± 9.6	4.8 ± 2.3	Aerobic exercise	30 min	1–2 times/wk	19 weeks	60–80% HRmax	92	Events: 22	Total: 92	90	Health education	Events: 18	Total: 90	A2
21	MARCUS	1995	20 (0/20)	37.5 ± 8.9	-	Aerobic exercise	30–45 min	3 times/wk	15 weeks	70–85% HRmax	10	Events: 3	Total: 10	10	Health education	Events: 1	Total: 10	A1
22	Oncken	2020	301 (0/301)	55.8 ± 6.2	5.3 ± 1.9	Aerobic and resistance exercise	60 min	2 times/wk	24 weeks	50–69% HRmax	150	Events: 30	Total: 150	151	Relaxation	22	151	A1
23	Van Rensburg	2013	162 (107/55)	30.8 ± 9.8	4.8 ± 1.9	Treadmill exercise	20 min	–	–	(1) 40% HRR	48	1.33	9.560769	48	Watch video	−2.18	9.866707	D1
												−1.62	9.560769			−0.5	5.804369	D2
										(2) 75% HRR	66	2.05	9.370011	48		−2.18	9.866707	D1
												−1.54	5.721678			−0.5	5.804369	D2
24	Linke	2012	38 (15/23)	43.6 ± 11.5	5.2 ± 2.3	Multi_x0002_component exercise	60 min	–	12 weeks	RPE 12–14	19	Events: 3	Total: 19	19	Internet-based smoking cessation program	Events: 3	Total: 19	A1
25	Williams	2010	60 (0/60)	42.37 ± 11.55	4.82 ± 2.52	Aerobic exercise	40–65 min	3 times/wk	12 weeks	64–85% HRmax	30	Events: 14	Total: 29	30	Health education	Events: 7	Total: 30	A1
												10	29			6	30	A2
26	Bize	2010	481 (272/209)	42.4 ± 9.7	5.4 ± 2.2	Aerobic exercise	45 min	1 time/wk	9 weeks	40–60% VO2max	229	Events: 107	Total: 299	252	Health education	115	252	A2
												−2	7.9		0.1	7.9		D2
27	Ciccolo	2011	25 (12/13)	36.5 ± 12.0	4.0 ± 2.6	Resistance exercise	60 min	2 times/wk	12 weeks	65–75% RM	13	6	13	13	Watch video	2	12	A1
												2	13			1	12	A2
28	Oh	2014	23 (15/8)	23.96 ± 4.83	2.78 ± 1.78	Cycling	15 min	–	–	(1) 40–50% HRR	14	−1.74	1.155725	23	Sitting passively	0.09	1.332817	B2
										(2) 70–75%HRR	23	−1.74	1.091238	23		0.09	1.332817	
29	Masiero	2020	50 (24/26)	23.83 ± 3.65	4.00 ± 1.41	Cycling	10 min	–	–	Moderate intensity	50	−0.56	0.6226355	50	–	−0.71	0.355106	B1
30	Jeffries	2020	55 (34/21)	28.16 ± 10.4	2.98 ± 2.01	Yoga	30 min	–	–		25	−1.36	1.938143	30	Read	−0.44	1.713505	B1
31	Tritter	2015	30 (10/20)	40.19 ± 10.30	4.53 ± 2.27	Treadmill exercise	15 min	–	–	45–68% HRR	15	−2.74	1.306637	15	Sitting quietly/ Read	−2.2	1.45712	B1
32	Schneider	2014	48 (14/34)	42.63 ± 13.38	4.22 ± 1.93	Treadmill exercise	10 min	–	–	40–68% HRR	23	−1.95	1.6668233	25	Sitting passively	−0.17	0.6794851	B1
33	Van Rensburg	2009	20 (15/5)	29.05 ± 9.37	4.0 ± 2.5	Stationary cycling	15 min	–	–	RPE 11–13	23	−0.8	1.3114877	23	Sitting passively	0.3	1.1031319	B1
34	VanRensburg(a)	2008	23 (15/8)	23.1 ± 4.6	3.4 ± 2.03	Walk on treadmill	15 min	–	–	RPE 10.8 ± 1.67	20	−1.9	1.374733	20	Sitting passively	0.4	1.053043	B1
35	Prapavessis	2007	121 (0/121)	38.0 ± 11.7	-	Aerobic exercise	45 min	3 times/wk	12 weeks	60–75% HRmax	68	Events: 46	Total: 68	53	Health education	40	53	A1
												40	68			36	53	A2
36	Van Rensburg	2012	20 (−)	18–50	2.3 ± 1.3	Stationary cycling	10 min	–	–	RPE 11–13	20	−1.95	2.013082	20	Sitting passively	−0.35	1.430909	B1
												−1.3	1.552417			0.3	1.479865	B2
37	Everson	2008	45 (25/20)	21.8 ± 2.2	3.36 ± 1.89	Cycling	10 min	–	–	(1) 40–59% HRR	15	−1.92	1.68961534	15	Sitting passively	0.51	0.42	B2
												−0.41	1.3622041			0.64	1.33292911	C5
										(2) 60–84% HRR	15	−1.81	1.33315415	15		0.51	0.42	B2
												−0.63	1.32317799			0.64	1.33292911	c5
38	Marcus	1999	281 (0/281)	40.2 ± 8.96	6.1 ± 2.0	Aerobic exercise	30–40 min	3 times/wk	12 weeks	60–85% HRR	134	Events: 41	Total: 134	147	Health education	32	147	A1
												16	109			12	108	A2
39	Allen	2018	32 (12/20)	30.3 ± 1.0	–	High-intensity interval training (HIIT)	20 min	–	12 weeks	80–90%HRR	21	B1: 0.25	B1: 0.31	11	–	B1: 0.5	B1: 0.27	B1, C4, D1, D2
												C4: 0.50	C4: 1.09					
												D1: 0.63	D1: 1.08			C4: 1.50	C4: 0.93	
												D2: 2.88	D2: 1.39					
						Continuous aerobic (CA) exercise	30 min	–	12 weeks	–		B1: 0.60	B1: 0.51			D1: 0.10	D1: 1.68	
												C4: 4.00	C4: 3.05					
												D1: 1.60	D1: 1.78			D2: 2.70	D2: 1.56	
												D2: 4.40	D2: 2.06					
40	Jeffries	2018	55 (34/21)	28.16 ± 10.40	2.98 ± 2.01	Yoga	30 min	2 times/wk	–	–	25	3.4	2.1	30	Read	4.83	1.84	B1
41	Abrantes(b)	2014	61 (23/38)	47.1 ± 8.5	5.9 ± 2.1	Aerobic exercise	20 min	–	12-week	55–69% HRmax	30	c	A1:	31	Health education	A1:	A1:	A1, A2, B1, C1, D1, D2
												A2:	A2:			A2:	A2:	
												B1: 3.5	B1: 1.2			B1: 3.9	B1: 1.7	
												C1: 4.7	C1: 1.2			C1: 5.7	C1: 1.7	
												D1: 1.1	D1: 0.2			D1: 1.2	D1: 0.3	
												D2: 4.1	D2: 0.8			D2: 3.7	D2: 0.8	
42	Bernard	2015	70 (29/41)	48.5 ± 10.9	6.3 ± 1.5	Aerobic exercise	40 min	–	8-week	60–85 HRmax	35	3.87	2.89	35	Health education	4.83	3.78	C4
43	Bize	2014	481 (272/209)	42.2 ± 10.0	-	brisk walking and slow jogging	45 min	4 times/wk	9 weeks	–	229	−0.2	7.9	252	Health education	0.1	7.9	C4
44	Klinsophon	2022	43 (23/20)	36.6 ± 10.9	-	breathing exercise	10 min	–	12 weeks	–	23	A1: Odd	A1: Odd	20	–	A1: Odd	A1: Odd	A1, A2, B1, D1, D2
												A2: Odd	A2: Odd			A2: Odd	A2: Odd	
												B1: 2.6	B1: 2.0			B1: 2.4	B1: 1.8	
												D1: 18.6	D1: 6.4			D1: 17.7	D1: 6.2	
												D2: 35.8	D2: 9.0			D2: 36.9	D2: 7.0	
45	Li	2022	20 (0/20)	45.0 + 15.0	–	RNC	4 mg per cigarette	–	12	–	7	B1: 2.4	B1: 0.4	7	Smoking research tobaccos	B1: 2	B1: 0.9	B1, C1, C2, C3, C5, C6, D1
												C1: 1.5	C1: 0.4					
												C2: 1.9	C2: 0.6			C1: 1.8	C1: 0.9	
												C3: 2.6	C3: 0.6					
												C5: 2.2	C5: 1.1			C2: 1.5	C2: 0.9	
												C6: 2.5	C6: 0.5					
												D1: 1.6	D1; 0.9			C3: 2.2	C3: 1.0	
						RNC + exercise	60 min	3/week (2/week)	12	75–85%HRmax	6	B1: 1.8	B1: 0.9					
												C1: 1.9	C1: 1.0			C5: 1.9	C5: 0.8	
												C2: 1.3	C2: 0.5					
												C3: 2.0	C3: 0.9			C6: 2.1	C6: 0.8	
												C5: 1.7	C5: 0.8					
												C6: 2.0	C6: 0.8			D1: 1.4	D1: 0.6	
												D1: 1.9	D1: 0.5					
46	Saritoy	2023	54	18–45	5.95 ± 2.39	MIA	45 min	3/week	8 weeks	40%HRmax	18	B1: 13.11	B1: 7.01	19	Care as usual	B1: 14.68	B1: 3.71	B1, C1, C6
												C6: 13.11	C6: 7.01					
												C1: 4.33	C1: 1.91			C1: 3.95	C1: 1.75	
					3.55 ± 1.85	MoIA				60%HRmax	17	B1: 14.12	B1: 7.02					
												C6: 14.12	C6: 7.02			C6: 12.58	C6: 6.53	
												C1: 3.53	C1: 1.37					
47	Stockton	2023	392 (149/243)	18–65	–	Physical activity intervention	–	150 min/week	12 months	Moderate-to-vigorous activity	199	D2: 3.91	D2: 0.79	193	Wellness Intervention	D2: 3.86	D2: 0.87	D2
												D1: 4.38	D1: 0.73			D1: 4.24	D1: 0.75	D1

A1: 7-Day Point Prevalence Abstinence (PPA) Rate; A2: Continuous Abstinence Rate; B1: Desire to Smoke (Dts); B2: Strength of Desire to Smoke (SoD); C1: Sleep Quality (SQ); C2: Depression (Dep); C3: Irritability (Irr); D1: Positive Emotions; D2: Negative Emotions.

### 3.3 Results of bias assessment

**Figure 3:**
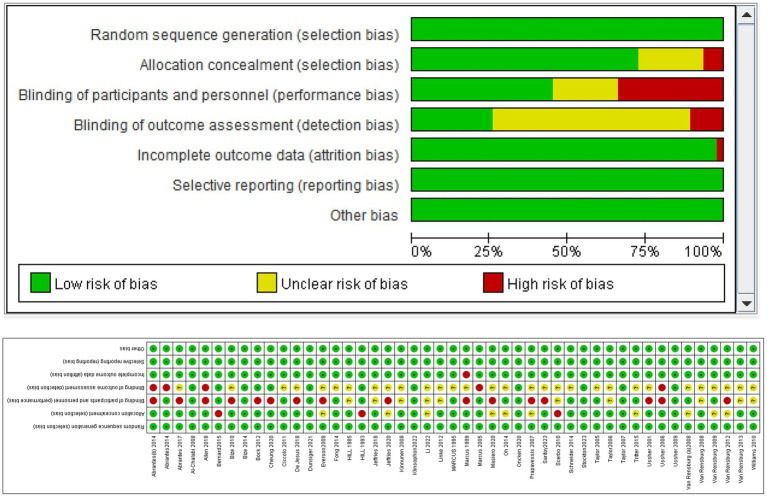
Results of bias assessment **(A,B)**.

Figure 3 summarizes the risk of bias assessment for the included studies. All 47 included studies employed random assignment and did not selectively report their research findings. However, due to the objective nature of exercise interventions, it was not feasible to maintain double-blinding among participants. Consequently, most researchers chose to inform participants of the intervention or did not conceal it, and specific details regarding blinding were not described, leading to a high risk of bias. Some of the included data had missing information, with some providing corresponding explanations, but a few still posed a high risk (Figures 3A,B).

### 3.4 Meta-analysis results

#### 3.4.1 Forest plot

##### 3.4.1.1 7-day point prevalence abstinence (PPA) rate (A1) and continuous abstinence rate (A2)

**Figure 4:**
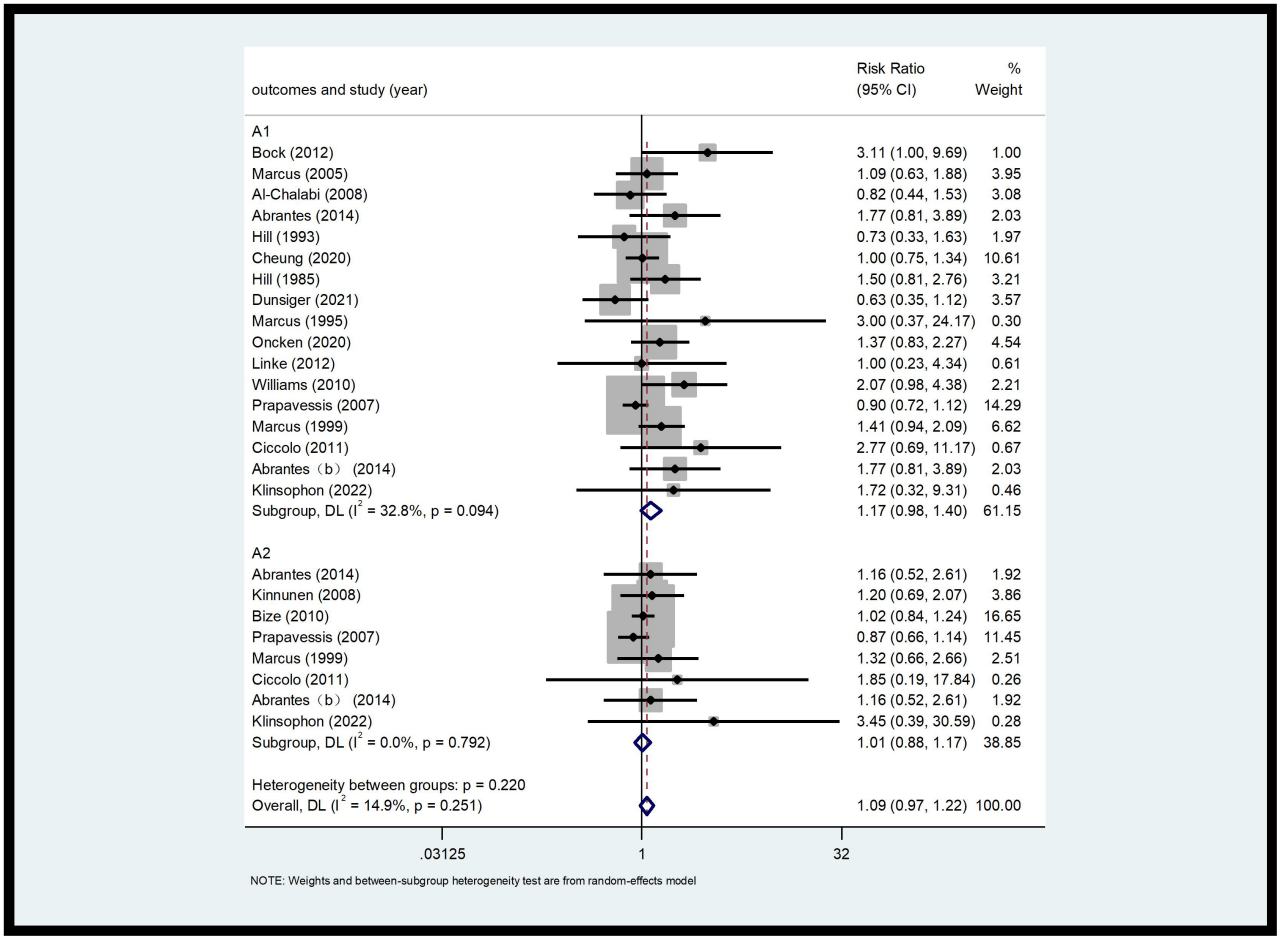
Forest plot of A1 and A2.

This analysis included 26 RCTs with a total of 2,902 participants (9, 17–29). The 7-day PPA rate (A1) and continuous abstinence rate (A2) were analyzed. As shown in Figure 4, the 7-day PPA rate (A1) had an *I*^2^ of 32.8%, indicating low heterogeneity. A fixed-effects model was used for analysis, with an RR of 1.17 and a 95% CI of 0.98, 1.40. For the continuous abstinence rate (A2), the *I*^2^ was 14.9%, also indicating low heterogeneity. Using a fixed-effects model, the RR was 1.01 with a 95% CI of 0.88, 1.17. Compared to the control group, the differences were not statistically significant (*p* > 0.05) (Figure 4).

##### 3.4.1.2 Desire to smoke (B1) and strength of desire to smoke (B2)

Two Likert scales were used to assess smoking urge: Desire to Smoke (B1) and Strength of Desire (B2). Regarding B1, a total of 14 RCTs (involving 904 participants, with 468 in the exercise group and 436 in the control group) were included in the assessment. For B2, a total of 10 RCTs (involving 512 participants, with 257 in the exercise group and 255 in the control group) were included in the assessment (30–45).

**Figure 5:**
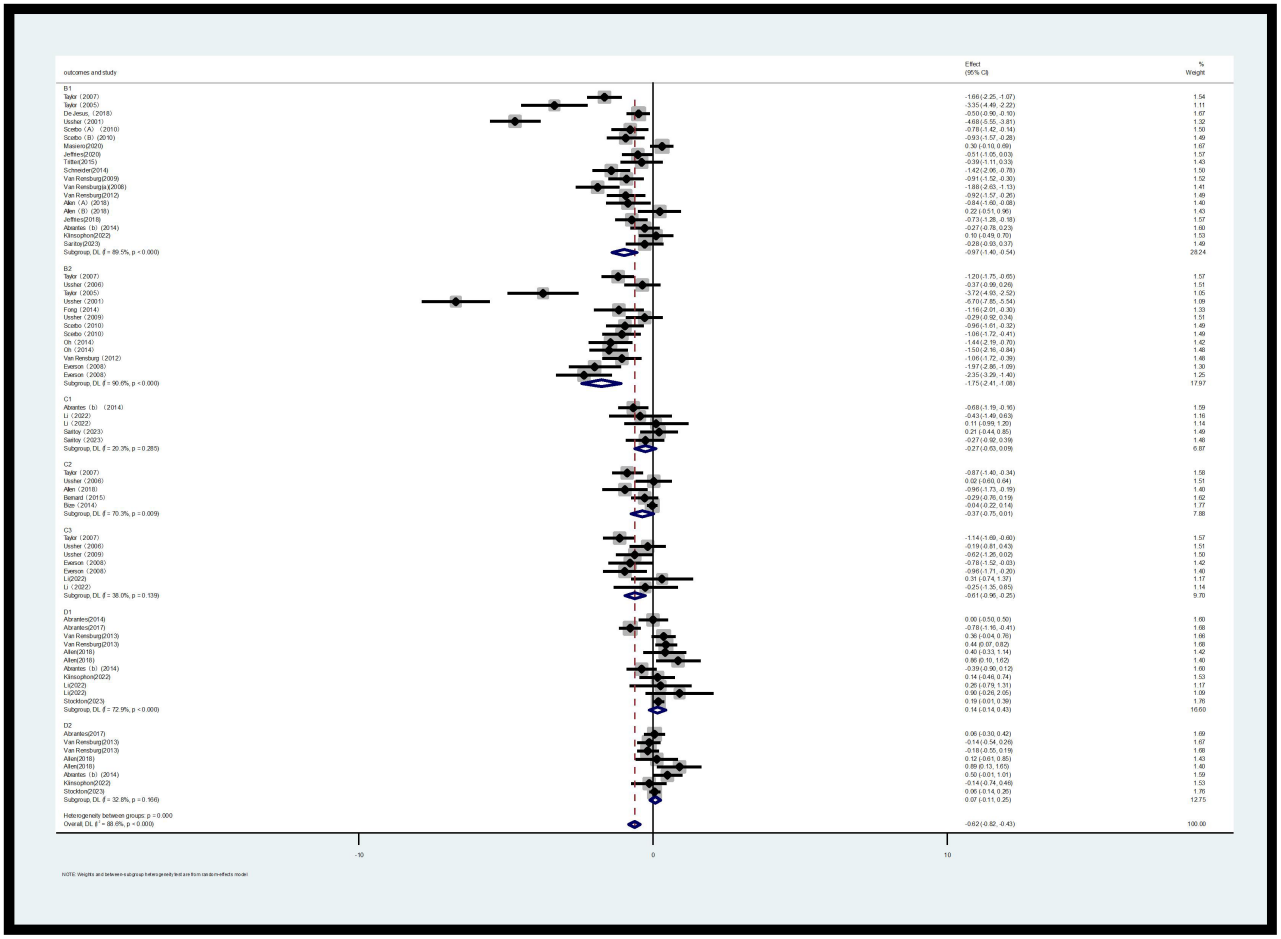
Forest plot of B1, B2, C1, C2, C3, D1, and D2.

The meta-analysis revealed that, based on the random-effects model, the standardized mean difference (SMD) for B2 was-0.97 with a 95% confidence interval of −1.40 to −0.54, and *p* < 0.000, which was statistically significant. This indicates that acute exercise can significantly reduce the desire to smoke among smokers. Compared to the control group, the effect of acute exercise interventions on the strength of desire to smoke was also significant, with an SMD of −1.75 and a 95% CI of −2.41 to −1.08, *p* < 0.000 (Figure 5). Additionally, heterogeneity tests showed high levels of heterogeneity for both B1 (*I*^2^ = 89.5%, *p* < 0.000) and B2 (*I*^2^ = 90.6%, *p* < 0.000).

##### 3.4.1.3 Sleep quality (C1), depression (C2) and irritability (C3)

This study employed three indicators to evaluate withdrawal symptoms: sleep quality (C1), depression (C2), and irritability (C3). Regarding the outcome indicator for sleep quality (C1), a total of 4 RCTs involving 148 participants (72 in the exercise group and 76 in the control group) were included in the assessment. For depression (C2), a total of 5 RCTs with 683 participants (336 in the exercise group and 347 in the control group) were included. Regarding irritability (C3), a total of 7 RCTs with 233 participants (108 in the exercise group and 125 in the control group) were included in the assessment (30, 31, 46–51).

As shown in Figure 5, for sleep quality (C1), the *I*^2^ was 32.9%, indicating low heterogeneity. The SMD for the exercise group was −0.30 with a 95% CI of −0.72 to 0.11. Compared to the control group, the difference was not statistically significant (*p* > 0.05). For depression (C2), the *I*^2^ was 70.3%, indicating high heterogeneity, and the difference compared to the control group was not statistically significant (*p* > 0.05). For irritability (C3), the *I*^2^ was 38.0%, and the SMD was −0.61 with a 95% CI of −0.96 to −0.21. Compared to the control group, the difference was statistically significant (*p* < 0.05), indicating that acute aerobic exercise can alleviate withdrawal symptoms of irritability to some extent.

##### 3.4.1.4 Positive emotions (D1) and negative emotions (D2)

This study investigated the emotions of smokers during the smoking cessation process, categorizing them into two broad domains: positive emotions and negative emotions. For negative emotions, a total of 11 RCTs involving 937 participants (487 in the exercise group and 450 in the control group) were included in the assessment. For positive emotions, a total of 8 RCTs involving 856 participants (444 in the exercise group and 412 in the control group) were included (9, 31, 47, 51–54).

As shown in Figure 5, the heterogeneity results indicate that for negative emotions, the *I*^2^ value is 72.9% (*p* < 0.000), suggesting a high level of heterogeneity. Therefore, a random-effects model was used for the analysis of positive emotions (note: this should likely be a clarification that the model choice was mentioned in the context of analysis overall, and not specifically for negative emotions as stated; however, following the instruction to ignore errors, we proceed with the translation as is). However, the presented results for negative emotions [standardized mean difference (SMD) = 0.14, 95% confidence interval (CI) (−0.14, 0.43), *p* < 0.000] indicate a statistical significance level that contradicts the statement that the difference is not statistically significant.

Meanwhile, for positive emotions, the *I*^2^ value is 32.8% (*p* = 0.166), indicating a lower level of heterogeneity. A fixed-effects model was used for analysis (if *I*^2^ < 50%, a fixed-effects model is adopted; otherwise, a random-effects model is used). The combined effect size shows an SMD of 0.06 with a 95% CI of −0.08 to 0.19 and *p* = 0.166. Compared to the control group, there is no significant difference in the impact on outcomes for the experimental group (Figure 5).

#### 3.4.2 Subgroup analysis

##### 3.4.2.1 Desire to smoke (B1) and strength of desire to smoke (B2)

The exercise intervention programs were grouped based on various factors, including the duration of each exercise session (<15 min, 15–30 min, and >45 min), the intensity of the exercise, sample size (<60 and ≥60), and the degree of tobacco dependence (<4.5 and ≥4.5). Since all the included studies focused on aerobic exercises, aerobic exercise type was considered as a covariate premise, and a random-effects model was used for subgroup analysis. The results of the subgroup analysis for B1 and B2 are presented in Table 2. According to the subgroup analysis, there were statistical differences in the combined results among the various subgroups (*p* < 0.005). However, the grouping factors were not identified as sources of heterogeneity.

**Table 2:** Results of subgroup analysis.

DtS SoD Dep	Heterogeneity test			Subgroups	Effect size and 95% CI	2-Tailed test		Number of studies	Sample size	Outcome indicators
	*X* ^2^	*p*	*I* ^2^			*Z*	*p*			
Intervention time	111.80	0.000	96.4%	<15 min	−0.697 (−0.927, −0.466)	−5.922	0.000	5	365	Dts
	109.77	0.000	95.4%		−1.359 (−1.667, −1.050)	−8.628	0.000	6	264	Sod
	4.58	0.032	78.2%		−0.50 (−0.90, −0.09)	−2.412	0.016	2	120	Dep
	41.32	0.000	78.2%	15–30 min	−0.910 (−1.107, −0.712)	−9.024	0.000	12	541	Dts
	17.66	0.007	66.0%		−1.334 (−1.599, −1.068)	−9.851	0.000	7	298	Sod
	–	–	–			–	–	–	–	Dep
	0.00	–	–	>30 min	−0.282 (−0.930, 0.366)	−0.854	0.393	1	37	Dts
	–	–	–		–	–	–	0	0	Sod
	5.87	0.053	65.9%		−0.11 (−0.27, 0.05)	−1.313	0.189	3	583	Dep
Intensity	50.91	0.000	90.2%	Low	−0.837 (−1.100, −0.57)	−6.257	0.000	6	271	Dts
	23.47	0.000	91.5%		−1.136 (−1.527, −0.745)	−5.691	0.000	3	130	Sod
	4.58	0.032	78.2%		−0.50 (−0.90, −0.09)	−2.412	0.016	2	120	Dep
	119.12	0.000	91.6%	Middle	−0.636 (−0.807, −0.466)	−7.330	0.000	11	633	Dts
	95.79	0.000	95.8%		−1.510 (−1.848, −1.171)	−8.745	0.000	6	241	Sod
	0.00	0.00	0.00		−0.04 (−0.22, 0.14)	−0.416	0.677	1	481	Dep
	0.03	0.868	0.0%	High	−0.890 (−1.382, −0.399)	−3.550	0.000	2	82	Dts
	6.18	0.186	35.3%		−1.336 (−1.661, −1.010)	−8.038	0.000	5	191	Sod
	2.16	0.141	53.8%		−0.47 (−0.87, −0.07)	−2.293	0.022	2	102	Dep
Sample sizes	75.37	0.000	81.4%	<60	−0.583 (−0.744, −0.423)	−7.121	0.000	14	645	Dts
	34.88	0.000	74.2%		−1.287 (−1.522, −1.052)	−10.734	0.000	10	384	Sod
	0.00	0.027	67.3%		−0.13 (−0.17, −0.19)	−2.452	0.014	1	32	Dep
	87.80	0.000	96.6%	≥60	−1.057 (−1.320, −0.793)	−7.852	0.000	4	298	Dts
	91.72	0.000	97.8%		−1.501 (−1.890, −1.112)	−7.557	0.000	3	178	Sod
	9.18	0.027	67.3%		−0.13 (−0.29, 0.02)	−1.673	0.094	4	671	Dep
Degree of tobacco dependence	70.49	0.000	85.8%	<4.5	−0.814 (−0.993, −0.634)	−8.883	0.000	11	574	Dts
	33.77	0.000	70.4%		−1.280 (−1.496, −1.064)	−11.616	0.000	11	438	Sod
	86.85	0.000	95.4%	≥4.5	−0.750 (−1.001, −0.498)	−5.839	0.000	5	305	Dts
	91.11	0.000	98.9%		−1.766 (−2.320, −1.213)	−6.253	0.000	2	124	Sod

##### 3.4.2.2 Depression (C2)

Based on the results of the subgroup analysis on depression (C2) according to the duration of each exercise session, the following findings were observed (Table 2). Comparison between the experimental group with 0–15 min of exercise and the control group showed an SMD of −0.44 with a 95% CI of −0.90 to −0.09 and *I*^2^ of 78.2%. The difference was statistically significant (*p* < 0.05). Comparison between the experimental group with >30 min of exercise and the control group showed an SMD of −0.30 with a 95% CI of −0.27 to 0.05 and *I*^2^ of 65.9%. The difference was also not statistically significant (*p* > 0.05). These results indicate that the duration of exercise is not a source of heterogeneity. Additionally, due to the limited number of studies assessing sleep quality and irritability as outcome indicators, insufficient data were available to conduct subgroup analyses for these factors (As shown in Table 2).

#### 3.4.3 Sensitivity analysis

##### 3.4.3.1 7-day point prevalence abstinence (A1) rate and continuous abstinence rate (A2)

**Figure 6:**
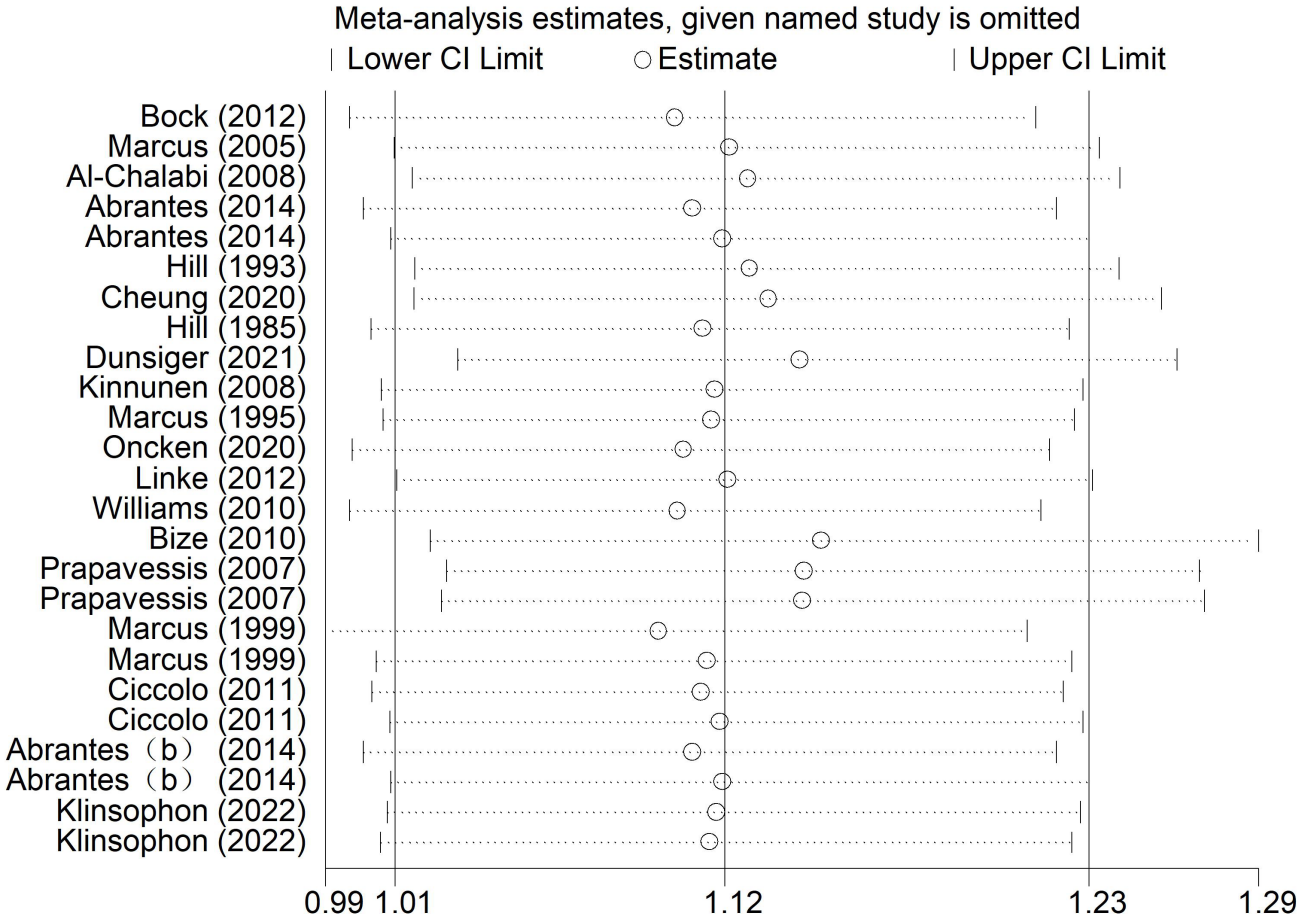
Results of sensitivity analysis for A1 and A2.

According to the results of sensitivity analysis, it was found that some of the studies might have low sample stability. Sensitivity analysis was further conducted using Stata 14 software to determine the stability and reliability of the samples in the included studies. As shown in Figure 6, the overall data tended to be stable, and the combined results were not influenced by any individual study. The sensitivity was low, ensuring the stability and reliability of the combined results in the subsequent meta-analysis (Figure 6).

##### 3.4.3.2 Desire to smoke (B1) and strength of desire to smoke (B2)

The results showed that for desire to smoke, the 95% confidence interval (CI) was (−1.40, −0.54), with an estimated median value of −0.97. For strength desire to smoke, the 95% CI was (−2.41, −1.08), with an estimated median value of −1.75. The overall data tended to be in a stable state, with the values in the graph fluctuating slightly around the median values of −0.97 (B1) or −1.75 (B2). Additionally, most of the confidence intervals tended to be negative, and the point estimates of the included studies were all within their respective confidence intervals. Therefore, the sensitivity analysis test showed that the results for desire to smoke (B1) and strength of desire to smoke (B2) were robust after excluding individual studies and conducting sensitivity analysis.

##### 3.4.3.3 Sleep quality (C1), depression (C2) and irritability (C3)

The results of sensitivity analysis showed that for sleep quality, the 95% confidence interval was (−0.63, 0.09), with an estimated median value of −0.27. For depression, the 95% CI was (−0.75, 0.01), with an estimated median value of −0.37. For irritability, the 95% CI was (−0.96, −0.25), with an estimated median value of −0.61. The overall data tended to be in a stable state, with the values in the graph fluctuating slightly around the median values of −0.27 (sleep quality), −0.37 (depression), or −0.61 (irritability). Additionally, the point estimates of the included studies were all within their respective confidence intervals. Therefore, the sensitivity analysis test showed that after excluding individual studies and conducting sensitivity analysis, the results for depression were robust.

##### 3.4.3.4 Positive emotions (D1) and negative emotions (D2)

**Figure 7:**
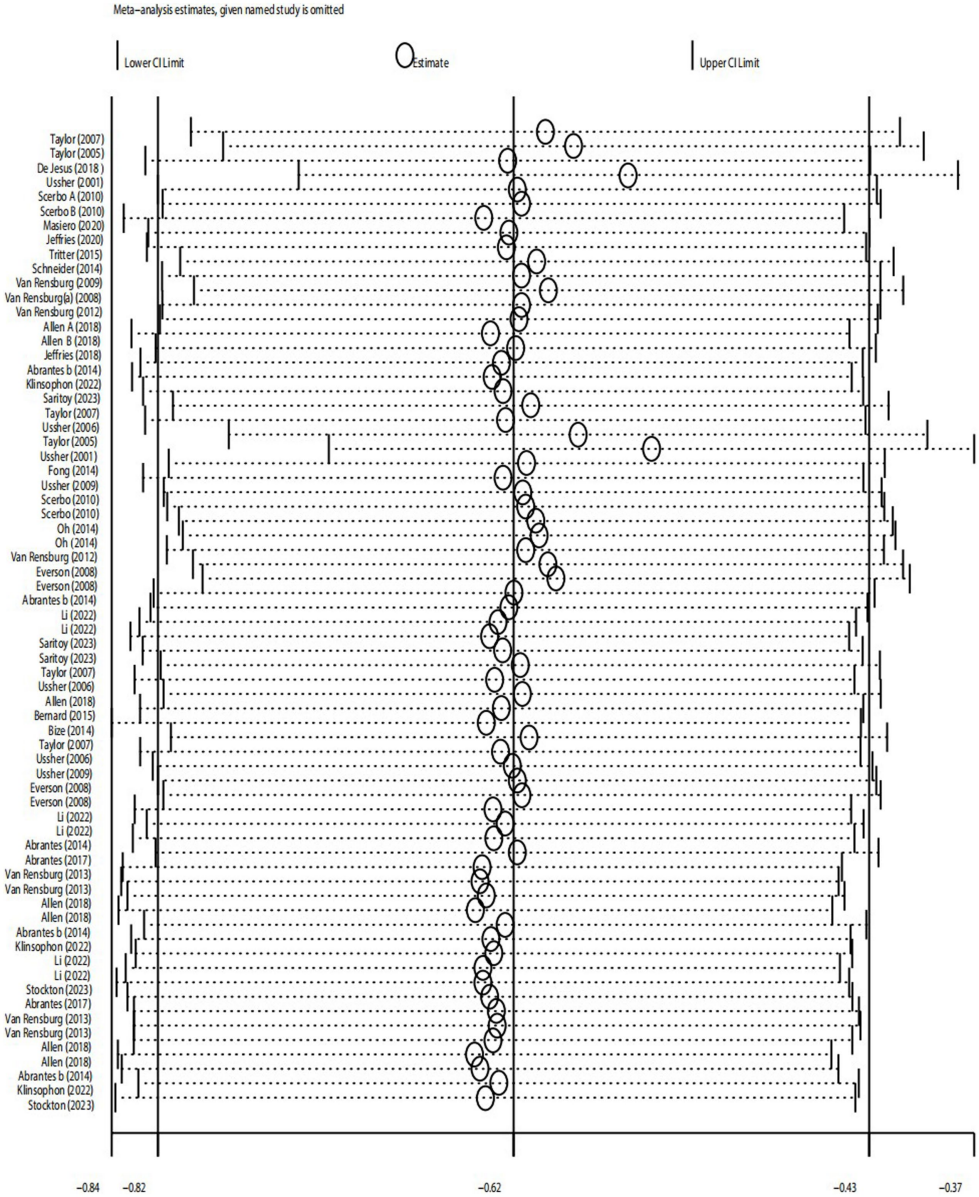
Results of sensitivity analysis for B1, B2, C1, C2, C3, D1, and D2.

The results showed that for negative emotions, the 95% confidence interval (CI) was (−0.14, 0.43), with an estimated median value of 0.14. For positive emotions, the 95% CI was (−0.11, 0.25), with an estimated median value of 0.07. Therefore, according to the sensitivity test, these two results were not robust. This suggests that the variability in the studies included in the analysis may have had a significant impact on the estimated effects of negative and positive emotions. Further investigation into the sources of heterogeneity and potential biases in the included studies is needed to improve the robustness of the results (Figure 7).

#### 3.4.4 Results of funnel plot

**Figure 8:**
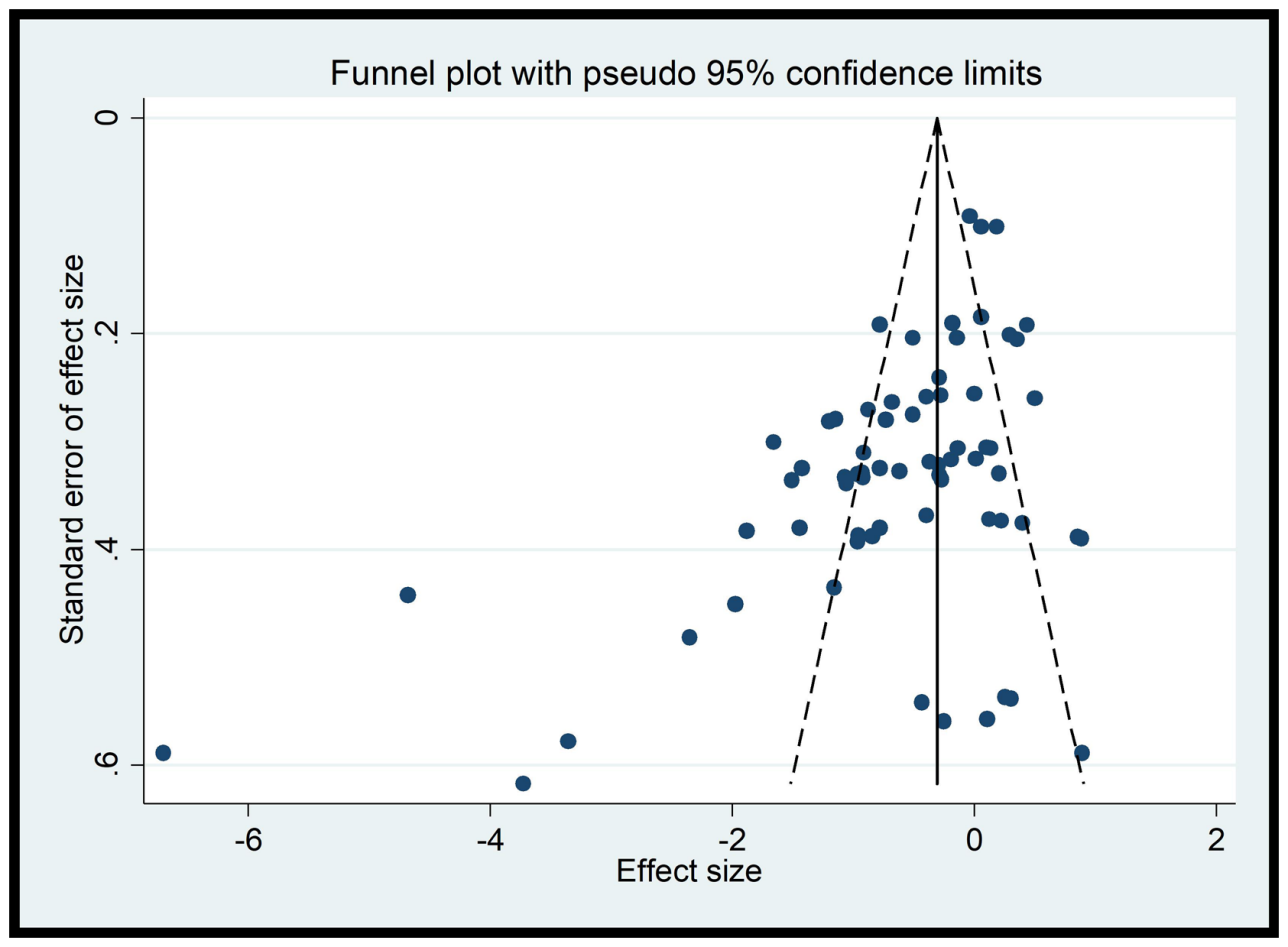
Results of funnel plot for B1, B2, C1, C2, C3, D1, and D2.

Since A1 and A2 were discussed as dichotomous variables separately from the others, they were not included in the funnel plot analysis for B1, B2, C1, C2, C3, D1, and D2. According to the funnel plots for these variables (Figure 8), a portion of the studies fall outside the 95% confidence interval, indicating the presence of high heterogeneity. Additionally, the funnel plots exhibit asymmetry with missing corners, suggesting the existence of publication bias. This may be due to the inclusion of low-quality studies with small sample sizes, which could lead to an overestimation of the combined effect, exaggerating the intervention effect, and causing bias.

To address these issues, further investigation into the sources of heterogeneity and potential biases in the included studies is necessary. This may involve conducting additional analyses, such as subgroup analyses or meta-regression, to explore the reasons for the heterogeneity and publication bias. Additionally, efforts should be made to identify and exclude low-quality studies or those with significant biases to improve the robustness and reliability of the results (Figure 8).

**Figure 9:**
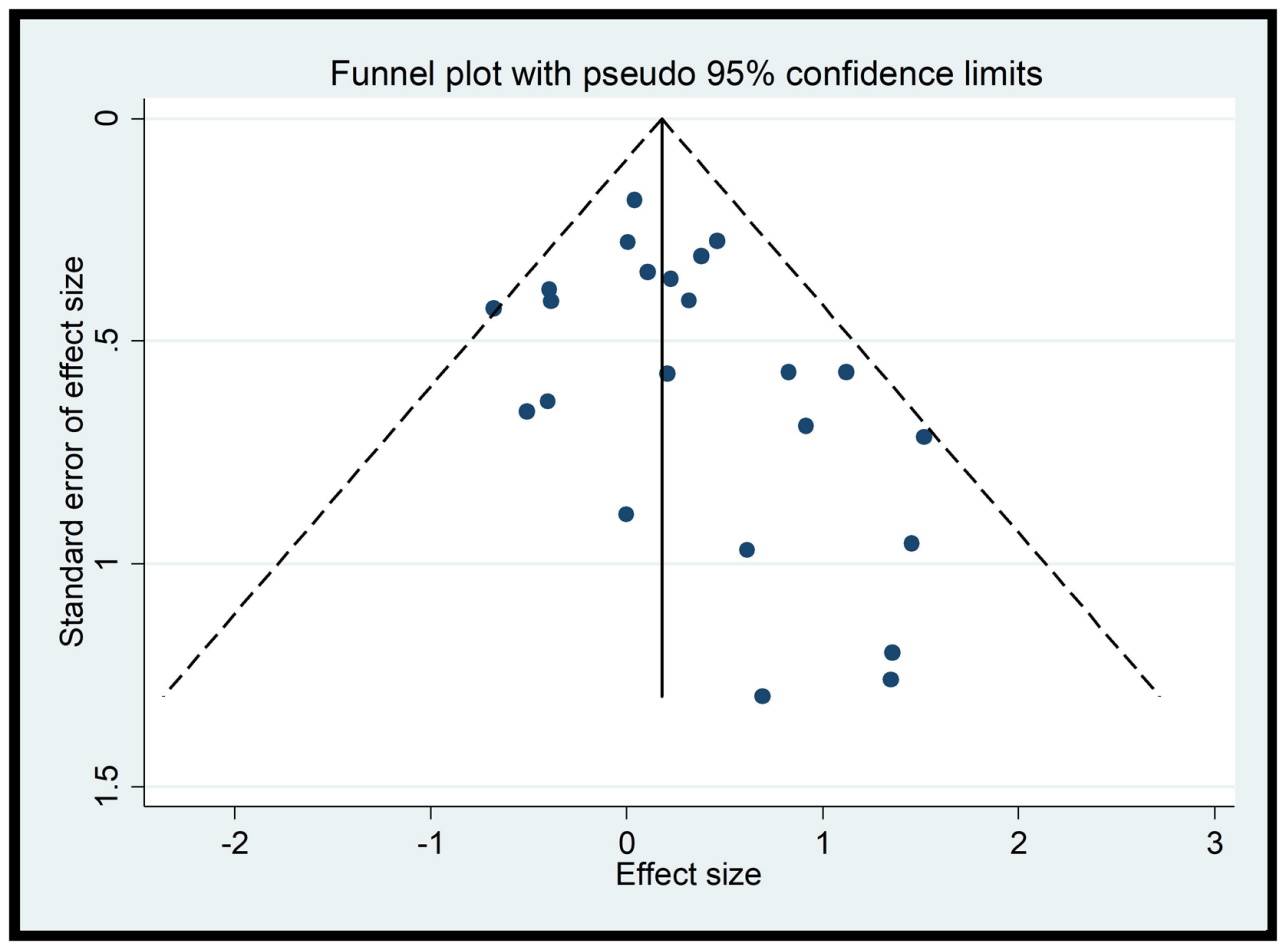
Results of funnel plot for A1, A2.

Consistent with the separate analysis for A1 and A2, the funnel plots for A1 and A2 reveal that most studies fall within the 95% confidence interval, indicating low heterogeneity (Figure 9). However, the funnel plots lack symmetry, suggesting the presence of publication bias. This could potentially be attributed to the inclusion of low-quality studies with small sample sizes, which may lead to an overestimation of the combined effect, exaggerating the intervention effect, and introducing bias (Figure 9).

#### 3.4.5 Results of Egger test

In this study, the experimental variables are B1, B2, C1, C2, C3, while D1 and D2 are continuous variables. Therefore, the Egger test was used to assess publication bias. The Egger test results indicate a potential for publication bias in B1 (DtS) and B2 (SoD) with *p*-values less than 0.05, suggesting that the results for these variables may be influenced by the presence of unpublished or underreported studies. On the other hand, the results for C1, C2, C3, D1, and D2 show no significant publication bias with *p*-values greater than 0.05, indicating that the available studies for these variables may be more representative of the true effect.

It is important to note that the presence of publication bias can affect the reliability and validity of the research findings. Therefore, when interpreting the results of this study, readers should consider the potential impact of publication bias on the B1 and B2 variables. Additional efforts may be needed to identify and include all relevant studies, especially those that may have been overlooked or underreported, to obtain a more accurate and unbiased estimate of the true effect.

## 4 Discussion

In our study, we aimed to comprehensively analyze trial results using both dichotomous outcomes (including 7-Day Point Prevalence Abstinence Rate and Continuous Abstinence Rate) and continuous variables (encompassing Desire to Smoke, its Strength, sleep quality, depression, irritability, positive emotions, and negative emotions). Notably, the literature frequently employs the Risk Ratio (RR) to interpret outcomes related to these dichotomous variables (55) particularly the 7-day PPA and continuous abstinence rates, prompting us to discuss 7-Day Point Prevalence Abstinence Rate and Continuous Abstinence Rate separately.

Smoking desire (intensity) and withdrawal symptoms are crucial indicators for assessing tobacco dependence (56). Higher smoking desire leads to shorter intervals between cigarettes and more pronounced withdrawal symptoms, which are major relapse triggers. Previous research suggests that physical exercise increases the time until the first cigarette following exercise (57). Thus, using exercise to alleviate these symptoms is crucial (58). Our study confirmed that acute aerobic exercise reduces tobacco cravings and withdrawal symptoms among smokers trying to quit. Specifically, the desire to smoke decreases significantly immediately following exercise (10). However, long-term exercise does not significantly impact tobacco dependence. Additionally, exercise intervention positively influences smokers or those attempting to quit, generating certain positive emotions.

Subgroup analyses to explore heterogeneity sources were restricted by original study designs. After examining individual articles, we found that excluding Tritter (45) and Taylor (32) led to more consistent sample characteristics and reduced methodological differences, suggesting they were main sources of high heterogeneity. The former had specific subject and control group limitations, while the latter focused on temporary smoking cessation and had unclear intervention criteria.

Exercise intensity is a significant control condition (59). Our subgroup analysis found that low, medium, and high-intensity exercise can all reduce tobacco dependence and craving to some extent. Especially, moderate to high-intensity acute aerobic exercise is the most effective intervention, and smokers with high tobacco dependence may benefit from increased exercise intensity. High-intensity exercise can significantly reduce tobacco dependence in a short period, especially for smokers with high dependence (13, 60). However, low-intensity exercise is ineffective in reducing smoking desire and intensity.

Another key finding from subgroup analyses, which specifically focused on withdrawal symptoms, reveals that exercise has a significant positive impact on reducing irritability among smokers attempting to quit. This finding aligns with previous research suggesting that exercise can alleviate negative mood states often experienced during withdrawal (13). However, our results indicate that the combined effect size for the intervention effects of exercise on depression is not statistically significant in our study population. To further investigate the sources of heterogeneity, we conducted additional subgroup analyses for depression. Notably, we found that only short-duration exercise (less than 15 min) has a positive effect on depression. Besides, our results also indicate that the intervention effect of exercise on sleep quality is not statistically significant. Our findings have important implications for future research and practice. While exercise may be beneficial in reducing irritability and, under certain conditions (such as short durations), depression, it may not have a significant impact on these outcomes for all smokers or across all durations of exercise. Therefore, practitioners should assess smokers’ specific needs and tailor interventions accordingly. This personalized approach can enhance the effectiveness of quit-smoking programs and improve overall outcomes for smokers seeking to overcome their tobacco dependence.

Notably, acute aerobic exercise has been identified as significantly effective in reducing cravings and withdrawal symptoms among smokers. While previous research acknowledged the immediate benefits of acute exercise on cigarette cravings, withdrawal symptoms, and smoking behavior (61), it did not delve deeply into the long-term effects of exercise or provide a direct comparison between acute and long-term interventions. The present meta-analysis evaluates both acute and long-term aerobic exercise interventions in smoking cessation, contributing to a more comprehensive understanding of the effects of exercise intensities and durations on tobacco dependence.

Our findings suggest that engaging in physical activity may positively influence smoking cessation attempts, potentially by providing a means to divert attention away from smoking cues and cravings. This aligns with previous research, such as the study by Hatzigeorgiadis et al. (62), which examined the acute effect of exercise on smoking delay among smokers. Notably, their results indicated that the use of self-regulation strategies, such as goal setting, could enhance the beneficial impact of exercise on reducing the urge to smoke immediately following physical activity (62–64). These strategies may facilitate the translation of the temporary relief from smoking cravings induced by exercise into more sustained smoking abstinence. Future research should explore the combined effects of exercise and self-regulation strategies on smoking cessation outcomes, potentially identifying optimal combinations and protocols that maximize the beneficial impact of these interventions.

Furthermore, compared to previous studies, the data in the present study is more comprehensive and up-to-date. It synthesizes data from 47 studies, including 57 randomized controlled trials, involving 4,267 participants. This larger sample size and more comprehensive dataset allow for a more robust analysis and more reliable conclusions.

This study has limitations. Firstly, blinding participants in exercise interventions is challenging, thereby presenting a significant risk of bias. Furthermore, incomplete outcome data further contributes to this bias. Additionally, some assumed and ignored calculations may have influenced our results. For instance, in the design of the experimental and control groups, it was challenging to ensure that the exercise intervention was the sole variable of difference. In some cases, participants were explicitly instructed not to smoke during the intervention period, which could potentially affect the test results by introducing an additional variable that was not fully accounted for in our analysis. Secondly, intervention specifics, such as intensity, were often incompletely reported, and exercise types lacked a consistent classification system. Lastly, future research should consider a wider range of outcome measures to comprehensively assess the effects of exercise on smoking behavior. For example, although emotion serves as a pivotal moderating variable, only a few studies explicitly outlined emotion classification criteria, which constrained the inclusion of emotion-related indicators in our analysis. To enhance the accuracy of future research, it is recommended to implement allocation concealment, ensure assessor blinding, augment the sample size, uphold data integrity, thoroughly consider and validate all relevant calculations, and carefully design experimental and control groups to minimize the impact of confounding variables.

## Data Availability

The original contributions presented in the study are included in the article/supplementary material, further inquiries can be directed to the corresponding author.
